# Before attributing a positive effect to levetiracetam extended compared to immediate release, appropriately designed studies are necessary

**DOI:** 10.12669/pjms.42.3.14882

**Published:** 2026-03

**Authors:** Josef Finsterer

**Keywords:** Epilepsy, Seizures, Levetiracetam, Psychiatric disease, Side effects

We read with interest the article by Özdemir et al. on a retrospective multicenter study of the effect of levetiracetam extended-release (LEV-ER) in 40 epilepsy patients who took LEV-ER either without prior use of levetiracetam immediate-release (LEV-IR) (n = 4) or with a history of taking LEV-IR (n = 36).^1^ Under LEV-ER, seizure frequency decreased from 1.6 seizures per month to 0.6 seizures per month.^1^ Psychiatric side effects under LEV-IR improved in 88% of patients who switched to LEV-ER.^1^ The study is interesting, but some points should be discussed.

The first point is the retrospective design of the study. Retrospective designs have several disadvantages. Since they rely on reviewing medical records that were not originally intended for research purposes, the data may be incomplete. Selection, classification, and recall errors also influence the results, and the reasons for differences in treatment between patients and those lost to follow-up cannot be determined, which may lead to bias.^2^ Furthermore, while retrospective designs can establish associations, they cannot prove causality, they do not guarantee control for confounding variables, and it can be difficult to form representative comparison groups, which is problematic for rare outcomes.^2^

The second point is that 70% of the patients included also took psychotropic drugs, but it was not specified what type of psychotropic drugs they were or what dosage they were administered.^1^ Since some psychotropic drugs can have an anticonvulsant effect (e.g., benzodiazepines, valproic acid), it is crucial to know the type of psychotropic drugs being taken. There are also psychotropic drugs that can trigger seizures, such as chlorpromazine and sulpiride.^3^ To what extent did the psychotropic drugs contribute to reducing or increasing the frequency of seizures and to reducing psychiatric side effects?

The third point is that no medications other than psychotropic drugs were reported.^1^ Since concomitant medications can significantly affect the absorption, metabolism, and excretion of ASM (e.g., warfarin, digoxin, oral contraceptives, probenecid), it is crucial to know all concomitant medications that the included patients took regularly.

The fourth point is that no comorbidities other than psychiatric disorders were reported. Since heart disease, renal insufficiency, or liver disease can significantly affect the metabolism and excretion of levetiracetam metabolites, it is important to know how many patients suffered from heart, liver, or kidney disease.

The fifth point relates to the one-third of patients who took not only LEV-ER but also other ASMs (Table-I).^1^ What type of ASMs did patients on polytherapy take, and at what dosage? Was the dosage or type of ASMs other than LEV-ER changed during the observation period? In patients receiving polytherapy with ASMs, it is difficult to determine which of the ASMs or which combination of ASMs was truly beneficial.

The sixth point is that medication adherence was not adequately monitored.^1^ None of the included patients had their adherence to LEV-ER monitored by measuring LEV blood levels. If adherence is based solely on the patient’s statement, it can be quite inaccurate.

The eighth point is that the cause of epilepsy in the cohort was not clearly established.^1^ According to the 2017 ILAE classification,^4^ epilepsy is divided into focal, generalized, combined generalized and focal, and unknown epilepsy.^4^ We should know how many patients had these types of epilepsy. Overall, appropriately designed studies are needed before attributing a positive effect to LEV-ER compared to LEV-IR.
